# Novel approaches against epidermal growth factor receptor tyrosine kinase inhibitor resistance

**DOI:** 10.18632/oncotarget.24624

**Published:** 2018-03-08

**Authors:** Carina Heydt, Sebastian Michels, Kenneth S. Thress, Sven Bergner, Jürgen Wolf, Reinhard Buettner

**Affiliations:** ^1^ Molecular Pathological Diagnostics, Institute of Pathology, University Hospital Cologne, Cologne, Germany; ^2^ Center of Integrated Oncology Köln-Bonn, University Hospital Cologne, Cologne, Germany; ^3^ Department I of Internal Medicine, Center for Integrated Oncology Köln-Bonn, University Hospital of Cologne, Cologne, Germany; ^4^ Translational Science, Oncology iMED, AstraZeneca, Waltham, MA, USA; ^5^ Medical Affairs, AstraZeneca Oncology, Wedel, Germany

**Keywords:** epidermal growth factor receptor (EGFR), non-small-cell lung cancer (NSCLC), resistance, tyrosine kinase inhibitor (TKI), tumor heterogeneity

## Abstract

**Background:**

The identification and characterization of molecular biomarkers has helped to revolutionize non-small-cell lung cancer (NSCLC) management, as it transitions from target-focused to patient-based treatment, centered on the evolving genomic profile of the individual. Determination of epidermal growth factor receptor (*EGFR*) mutation status represents a critical step in the diagnostic process. The recent emergence of acquired resistance to “third-generation” EGFR tyrosine kinase inhibitors (TKIs) via multiple mechanisms serves to illustrate the important influence of tumor heterogeneity on prognostic outcomes in patients with NSCLC.

**Design:**

This literature review examines the emergence of TKI resistance and the course of disease progression and, consequently, the clinical decision-making process in NSCLC.

**Results:**

Molecular markers of acquired resistance, of which T790M and *HER2* or *MET* amplifications are the most common, help to guide ongoing treatment past the point of progression. Although tissue biopsy techniques remain the gold standard, the emergence of liquid biopsies and advances in analytical techniques may eventually allow “real-time” monitoring of tumor evolution and, in this way, help to optimize targeted treatment approaches.

**Conclusions:**

The influence of inter- and intra-tumor heterogeneity on resistance mechanisms should be considered when treating patients using resistance-specific therapies. New tools are necessary to analyze changes in heterogeneity and clonal composition during drug treatment. The refinement and standardization of diagnostic procedures and increased accessibility to technology will ultimately help in personalizing the management of NSCLC.

## INTRODUCTION

### Overview of EGFR TKI resistance

Non-small-cell lung cancer (NSCLC) accounts for 85–90% of primary lung cancers [1, 2]. The identification of specific molecular targets against which therapies for NSCLC can be directed has prompted a shift towards personalized treatment [3, 4] and, with this, improved survival rates [4, 5]. In the tumors of patients with NSCLC of adenocarcinoma histology, three out of four of the known driver gene mutations are targetable with regulatory approved, specifically targeted treatments; these are: activating mutations in the epidermal growth factor receptor (*EGFR*); activating translocations of anaplastic lymphoma kinase (*ALK*); rearrangements of ROS proto-oncogene 1, receptor tyrosine kinase (*ROS1*); and the kinase activating mutation V600E in the *BRAF* oncogene [5–7]. The most common *EGFR* mutations are present in the tumors of approximately 13–20% of Western and 40–48% of Asian patients with NSCLC of adenocarcinoma histology (corresponding data for non-adenocarcinoma: 3–5% and 8%, respectively) and whilst *EGFR* mutations may occur in any patient, they show a clear association with Asian ethnicity, female gender, and never-smoker status [6, 8–15]. For patients with *EGFR* mutation-positive NSCLC, first-line treatment with EGFR tyrosine kinase inhibitors (TKIs; specifically gefitinib [IRESSA^TM^], erlotinib [TARCEVA^®^], and afatinib [GIOTRIF^®^]) has been associated with superior objective response rates and progression-free survival compared with chemotherapy [5, 16–18].

The presence of *EGFR* mutations is the fundamental driver of response to EGFR TKIs [19–26]. However, most patients will acquire resistance to first-line EGFR TKIs and disease progression usually occurs within 6–24 months of treatment initiation [20, 22–24]. Unfortunately, the inevitability of acquired resistance keeps apace with new drug development, and resistance to second-line “third-generation” EGFR TKIs (e.g. osimertinib [TAGRISSO^TM^] and rociletinib) has also been reported [27–31]. The molecular mechanisms underlying resistance to first- and second-line EGFR TKI therapy are becoming increasingly clear. Molecular alterations triggering resistance may alter the drug target itself (e.g. the T790M resistance mutation in the kinase binding domain of EGFR, the most common mechanism of acquired resistance to first-line EGFR TKIs) or activate alternate signal transduction pathways (e.g. *MET* amplification).

### Tumor heterogeneity and development of resistance

Tumor heterogeneity — the presence of subclones of cells with distinct genotypes and divergent biologic behaviors — represents a key driver of cancer progression. This can include different cell subclones within a primary tumor (intratumor heterogeneity), between or within associated metastases (inter-/intra-metastatic heterogeneity), and between multiple tumors within an individual (intertumor heterogeneity) [32–37]. Tumor heterogeneity can be fostered by genomic instability [32] and genetically unstable cell subclones accumulate genetic alterations due to various kinds of selection pressure, including anti-neoplastic treatments and changes within the fluctuating microenvironment — a concept termed “tumor Darwinism” [32–34, 38]. Tumor stem cells may represent an important source of heterogeneity, as they have sufficient lifespan, and the proven capacity to self-renew and differentiate, which allows them to accumulate the genetic alterations necessary for treatment resistance [39]. Treatment resistance may also be due to failure of drug delivery; however, the mechanisms responsible for this are beyond the scope of this review.

**Figure 1:**
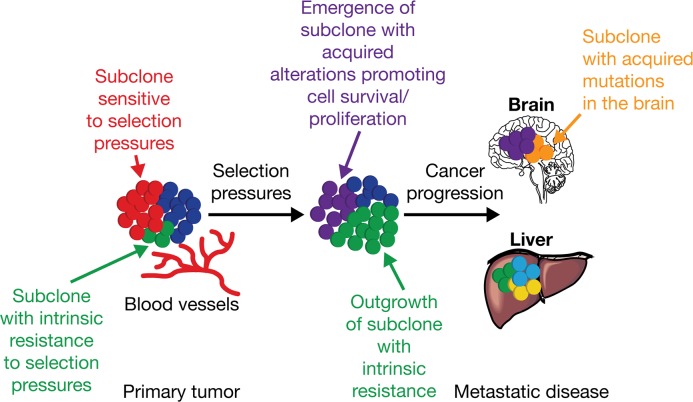
Intratumor heterogeneity and clonal evolution Adapted from Jamal-Hanjani M, Quezada SA, Larkin J, Swanton C. Translational implications of tumor heterogeneity. Clin Cancer Res 2015; 21: 1258-1266, with permission from AACR [34]. Primary tumors consisting of different subclones may be subjected to various selection pressures (e.g. chemotherapy, and micro-environmental factors such as hypoxia, and infiltrating stromal and immune cells). Under the influence of selection pressures, subclones with intrinsic resistance (*green*) can outgrow a tumor mass, potentially leading to disease progression, and/or can acquire somatic alterations (*purple*) promoting cell survival, proliferation, and metastatic tumor formation. The outgrowth of some subclones (*red*) may be constrained by selection pressures that they are sensitive to; for example, targeted therapy against a tumor subclone with a somatic alteration sensitive to therapy.

Drug treatment (e.g. EGFR TKIs, cytotoxic chemotherapy) can promote the selection of resistant clones and subclones with genetic aberrations that eventually drive treatment resistance and disease progression [33, 40–42] (Figure 1). For example, the increased prevalence of the T790M resistance mutation detected over time during first-line gefitinib treatment for NSCLC provides evidence for clonal expansion during EGFR TKI treatment [43].

**Figure 2:**
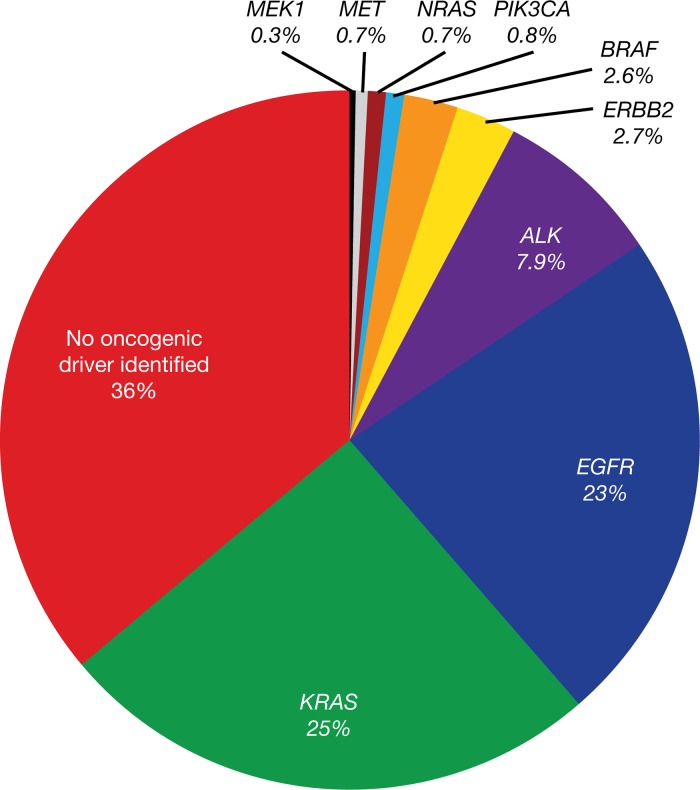
*EGFR* driver mutations identified in the Lung Cancer Mutation Consortium cohort (lung adenocarcinoma) Reprinted from Sholl LM, Aisner DL, Varella-Garcia M et al. Multi-institutional oncogenic driver mutation analysis in lung adenocarcinoma: the Lung Cancer Mutation Consortium experience. J Thorac Oncol 2015; 10: 768-777, with permission from Elsevier) [46].

Tumor heterogeneity, therefore, has the potential to vastly complicate the treatment process, given that it is difficult to anticipate and then selectively target multiple molecular changes in rapidly evolving disease [33, 44, 45]. This can confound the predictive accuracy of prognostic biomarkers, particularly when relying on historic tissue samples. However, the emergence of liquid biopsy, and advances in “real-time” analysis methodology, may eventually help to accurately track the evolution of the tumor and, in this way, optimize targeted treatment approaches. Understanding tumor heterogeneity in terms of disease progression and relating observations in the patient to changes happening at the molecular level is the key to effective disease management. In NSCLC, treatment decisions are currently made on the basis of “clinical” radiologic indicators of disease progression, denoting a worsening of tumor burden with the emergence of clinical symptoms. Acquired resistance leading to disease progression is often driven by the development of secondary mutations that can be verified following detection of genetic biomarkers. This review examines the emergence of TKI resistance and the impact of tumor heterogeneity on the clinical decision-making process in NSCLC.

## BIOMARKERS

The ongoing characterization of the key drivers of response and resistance to TKI therapies has allowed the identification of molecular biomarkers that may form the basis of diagnosis and personalized treatment for patients with NSCLC [3]. In this section we review some of the key biomarkers and consider their relevance at either the point of initial diagnosis or at clinical or molecular disease progression.

### Diagnostic biomarkers which can be used to guide treatment options

*EGFR* mutations (Figure 2) are important predictive biomarkers at diagnosis for the efficacy of first-line EGFR TKI treatment [46]. Determination of *EGFR* mutation status is, therefore, mandatory in the diagnosis of NSCLC, and should also be performed in squamous-cell lung carcinoma in never-smokers [2, 47–50]. For patients with *EGFR* mutation-positive NSCLC, EGFR TKI treatment is advocated, whereas chemotherapy or immunotherapy may be beneficial for patients with *EGFR* mutation-negative NSCLC [5, 19, 22].

**Figure 3:**
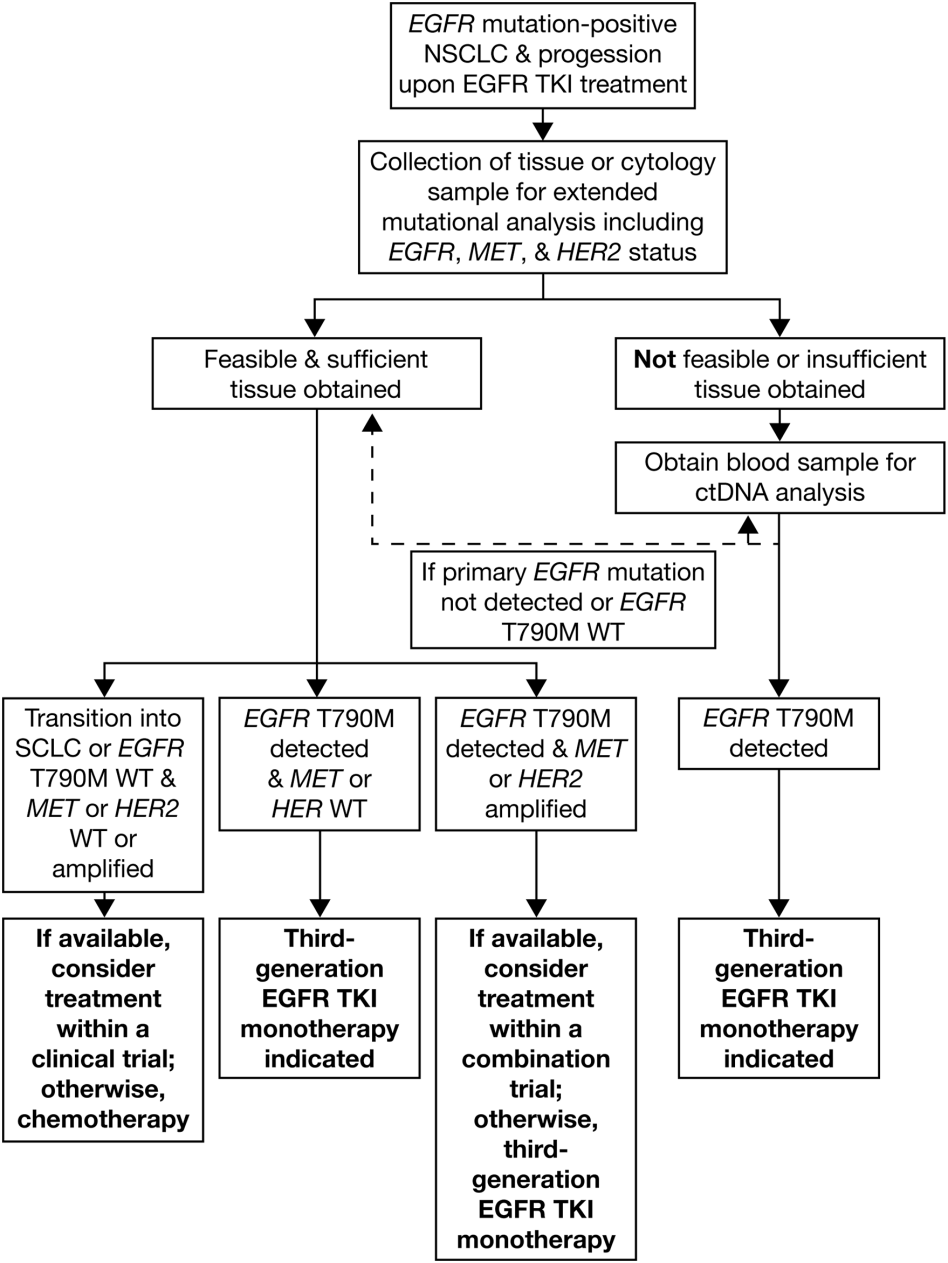
*EGFR* mutation testing algorithm WT, wild-type.

### Biomarkers which may indicate progression and may be used to guide subsequent treatment options

The nature of post-progression treatment should be tailored according to identified resistance mechanisms, as well as sites and the pace of disease progression [51]. Continued treatment beyond progression with concurrent local treatment in oligoprogressive disease when local treatment is feasible has been widely adopted in NSCLC [50]. The most recent National Comprehensive Cancer Network (NCCN) Clinical Practice Guidelines advocate the continued use of erlotinib, gefitinib, or afatinib in patients with asymptomatic progression, given that discontinuation of these EGFR TKIs has been associated with accelerated disease progression in terms of symptoms and tumor size [2, 50]. The basis for this post-progression prolongation of survival comes from the continued application of selective pressure on EGFR TKI-sensitive tumor subclones, thereby preventing regrowth and reducing the risk of rapid progressive disease once treatment is withdrawn [52]. The recommendation of treatment beyond progression may be based on prospective and retrospective analyses. Small retrospective studies of treatment beyond progression combined with local ablative therapy in patients with *EGFR* mutations or *ALK* translocations that experience oligoprogressive disease to TKI treatment have shown benefit in terms of progression-free survival and overall survival [53, 54]. The ASPIRATION study, to date the only prospective study to investigate the continuation of erlotinib beyond progression, shows benefit associated with continued treatment in select patients gaining a median 3.1 months of progression-free survival [55].

Whilst acquired resistance to EGFR TKIs may arise from multiple, complex mechanisms, several treatment strategies have been developed that specifically target the most frequent routes: *EGFR* T790M mutations, *MET* amplifications, and human epidermal growth factor receptor 2 (*HER2*) amplifications [56].

#### EGFR T790M

T790M mutations are secondary mutations in *EGFR* that are associated with acquired resistance to early-generation EGFR TKIs [57, 58]. Being the most common mechanism of acquired resistance, T790M mutations occur in approximately 50–60% of cases [56, 59] and are associated with impaired binding of the EGFR TKI to the tyrosine kinase domain of the EGFR [60, 61]. The T790M mutation increases the affinity of the binding pocket for ATP, thus interfering with the binding of EGFR TKIs and affecting specificity [61, 62]. The substitution is at a key site in the catalytic cleft of the EGFR TKI domain, located in the back of the ATP binding cleft. The amino acid substitution (threonine to methionine) leads to a bulkier side chain, resulting in steric hindrance which prevents the binding of reversible first-generation TKI molecules [62]. However, the T790M mutation itself does not interfere with ATP-binding and activation of EGFR, thus the tumor remains dependent on the EGFR pathway. This fact is important for further EGFR TKI treatment, as the T790M mutation remains sensitive to irreversible inhibitors. In contrast to the reversible inhibitors, the irreversible inhibitors overcome the resistance mechanism by covalent binding with Cys-797 in the ATP-binding cleft [61, 62].

As a collective drug class, the irreversible inhibitors are called “third-generation” EGFR TKIs and have shown potent and highly specific activity against T790M-mediated EGFR TKI resistance, with Phase I/II/III data in circulation (see Table 1 for a summary of efficacy outcomes only) [63–73].

**Table 1:** Summary of “third-generation” EGFR TKIs showing activity against acquired resistance mediated by T790M

“Third-generation” EGFR TKI	Study outcomes	Comment
**Jänne et al 2015** [63] **(AURA study)**		
Osimertinib	ORR (61% vs. 21%) and PFS (median 9.6 months vs. 2.8 months) improved in patients with *EGFR* T790M mutation-positive NSCLC following progression during prior EGFR TKI therapy, vs. patients with non-T790M-mediated resistance	Granted FDA accelerated approval for treatment of T790M mutation-positive NSCLC regardless of line of therapy (November 2015)
**Goss et al 2016** [64] **(AURA 2 study)**		
Osimertinib	Patients with *EGFR* T790M mutation-positive NSCLC showed early and durable objective response to osimertinib	Data support a potential change in clinical practice to evaluate tumors for the presence of *EGFR* T790M after progression
**Mok et al 2017** [65] **(AURA 3 study)**		
Osimertinib	In patients with *EGFR* T790M mutation-positive NSCLC, prolonged PFS (median 10.1 months vs. 4.4 months; HR 0.30; *P*<0.001) and higher ORR (71% vs. 31%; OR 5.39; *P*<0.001) observed with osimertinib vs. platinum plus pemetrexed therapy. PFS also prolonged with osimertinib vs. platinum plus pemetrexed therapy in patients with CNS metastases	Benefits of osimertinib observed in Phase II trial and confirmed in Phase III trial
**Sequist et al 2015** [66] **(TIGER-X study)**		
Rociletinib	Higher ORR (59%) in patients with *EGFR* T790M mutation-positive NSCLC vs. patients with T790M-negative disease (TIGER-X). Pooled TIGER-X/TIGER-2 data revealed lower rate of confirmed response (28–34%) [67]. Mature confirmed response rate (TIGER-X) 45% [68]	Clinical enrollment in all ongoing clinical studies terminated (2016)
**Park et al 2015** [69]		
Olmutinib (HM61713)	Preliminary study reports ORR 58.8% (*n*=34) for HM61713 (dose >650 mg). Partial responses (unconfirmed; *n*=10) and disease stabilization (*n*=13) also observed	Granted Breakthrough Therapy designation by FDA (December 2015). Phase I/II studies ongoing (NCT01588145)
**Tan et al 2015** [70]**; Jia et al 2016** [71]		
EGF816	Potent inhibition of the most common *EGFR* mutations – L858R, exon 19 deletion and T790M – *in vitro* and in patient-derived xenograft models. Antitumor activity observed against T790M mutation-positive NSCLC across all dose levels examined	Phase I/II studies ongoing (NCT02108964)
**Yu et al 2016** [72]		
ASP8273	Robust antitumor activity in patients with *EGFR* T790M mutation-positive NSCLC	Phase I, II, and III studies ongoing (NCT02113813; NCT02192697; NCT02588261)
**Wang et al 2016** [73]		
PF-06747775	Under investigation in patients with advanced NSCLC with *EGFR* mutations (exon 19 deletion or L858R ± T790M)	Phase I/II studies ongoing (NCT02349633)

CNS, central nervous system; FDA, Food and Drug Administration; HR, hazard ratio; OR, odds ratio; ORR, objective response rate; PFS, progression-free survival.

#### MET

Although high-level amplifications of the proto-oncogene *MET* are uncommon in previously untreated NSCLC (~3% [74]), *MET* amplifications have been detected in 5–20% of tumor samples from patients with acquired resistance following first-line EGFR TKI therapy, and have been implicated in tumor cell proliferation and survival [35, 59, 75–82]. Co-occurrence of both MET and T790M resistance mechanisms may be found in between 7–39% of patients [83–85]; however, *MET* amplification may also occur independently of the T790M mutation, thereby representing a clinically distinct therapeutic target [76, 79, 80, 86]. In a pre-clinical setting, a combination of MET inhibition and EGFR inhibition has been shown to restore sensitivity to EGFR TKIs [87, 88] and preliminary clinical studies are ongoing (see later section; Table 2) [87–96]. *MET* amplification has also been implicated as a mechanism of resistance to the “third-generation” EGFR TKI osimertinib [94, 97, 98].

**Table 2:** Summary of combinatorial treatment approaches currently under pre-clinical and clinical investigation

Treatment combination	Outcome
**Gibbons et al 2016** [89]	
Durvalumab plus gefitinib	Durvalumab plus gefitinib displayed encouraging activity in TKI-naïve NSCLC patients with sensitizing *EGFR* mutations and was generally well tolerated [Ongoing Phase I open-label study]
**Oxnard et al 2015** [90]**; Ahn et al 2016** [91]**; Yang et al 2016** [92] **(TATTON study)**	
Osimertinib plus durvalumab (anti-PD-L1 monoclonal antibody), savolitinib (MET inhibitor), or selumetinib (MEK 1/2 inhibitor)	Encouraging clinical activity profile of osimertinib combinations in *EGFR*-mutation-positive NSCLC patients; emergence of interstitial lung disease in combination patients warrants further investigation (durvalumab arm now discontinued) [Ongoing Phase Ib study]
**Janjigian et al 2014** [93]	
Afatinib plus cetuximab (antibody therapeutic)	Afatinib plus cetuximab displayed robust clinical activity and a manageable safety profile in resistant *EGFR*-mutant lung cancers with and without T790M mutations [Phase Ib study]
**Ou et al 2016** [94]	
Osimertinib plus crizotinib (MET inhibitor)	High level of *MET* amplification post-progression on osimertinib, transient symptomatic benefit following osimertinib plus crizotinib (MET inhibitor) [Case report]
**Nakagawa et al 2012** [87]	
WZ4002 (mutant-selective EGFR TKI) plus E7050 (mutant-selective MET TKI)	Suppression of growth of erlotinib-resistant tumors caused by gatekeeper T790M mutation, *MET* amplification, and *HGF* overexpression [Pre-clinical]
**Smit et al 2016** [95]	
Erlotinib with/without INC280 (cMET inhibitor) vs. platinum chemotherapy plus pemetrexed	Erlotinib with/without INC280 compared with platinum plus pemetrexed, in patients with EGFR TKI-resistant NSCLC due to *cMET* amplification (*EGFR* T790M-negative) [Ongoing Phase Ib/II study]
**Jia et al 2016** [96]	
EAI045 (allosteric inhibitor of drug-resistant EGFR mutants) plus cetuximab (antibody therapeutic)	EAI045 plus cetuximab effective in mouse models of lung cancer driven by EGFR(L858R/T790M) and by EGFR(L858R/T790M/C797S) [Pre-clinical]
**Nanjo et al 2013** [88]	
Afatinib or WZ4002 plus crizotinib (MET inhibitor)	Crizotinib plus afatinib or WZ4002 potently inhibited the growth of mouse tumors induced by EGFR TKI-resistant cell lines. High-dose crizotinib plus afatinib associated with severe side effects [Pre-clinical]

HGF, hepatocyte growth factor; PD-L1, programmed death-ligand 1.

#### HER2

Amplification of *HER2* has been detected with a frequency of 12–13% in patients with progressive disease following first-line EGFR TKI treatment [78, 99]. Furthermore, it has been postulated that *HER2* amplification is involved in the development of resistance to “third-generation” EGFR TKIs, such as osimertinib — in a case report, a patient who had acquired a T790M mutation after progression with second-line gefitinib then went on to develop resistance to osimertinib, which was associated with *HER2* amplification in the absence of a C797S mutation in *EGFR* [100]. Targeted treatment of *HER2* amplification has been disappointing so far in NSCLC [101, 102], although HER2-directed antibodies and TKIs are under evaluation [103].

#### MEK/ERK pathway

Activation of the mitogen-activated protein kinase (MEK)/extracellular signal-regulated kinase (ERK) pathway has been observed in cell lines treated with EGFR TKIs, resulting in resistance to EGFR TKI monotherapy [104]. In patients treated with “third-generation” EGFR TKIs, MEK/ERK activation has also been described by different mechanisms [80]. Combinations of “third-generation” EGFR TKIs with MEK TKIs are being explored in Phase I trials – for example, osimertinib plus selumetinib (as *in vitro* data show reconstitution of EGFR dependency upon MEK inhibition) [91, 104].

#### Other rare resistance mechanisms

Many other genetic aberrations have been described in the setting of acquired resistance, either alone or in combination with other resistance mechanisms, such as EGFR TKI resistance or *MET* and *HER2* amplification. These genetic aberrations may also contribute to disease progression, including somatic phosphatidylinositol-4,5-bisphosphate 3-kinase catalytic subunit alpha (*PIK3CA*) mutations [37, 105], seen in 1–5% of patients [56, 78, 106], and loss of phosphatase and tensin homolog (*PTEN*), which controls the phosphatidylinositol 3-kinase/protein kinase B (PI3K/Akt) signal pathway [106, 107]. An additional mechanism of acquired resistance in *EGFR* mutation-positive NSCLC is transformation to the small-cell lung cancer (SCLC) phenotype, which has been reported in several patient cases [36, 108, 109]. Although relatively uncommon, the transition is detectable by standard pathologic examination of tissue biopsies and patients may respond well to SCLC-specific chemotherapy. It is also important to consider that other factors — such as the tumor microenvironment [106] — may be associated with resistance, further complicating full elucidation of resistance pathways. Indeed, the mechanism of resistance may be unknown in as many as 18–30% of patients [56, 59].

## FOCUS ON *EGFR* MUTATION TESTING METHODS AT DIAGNOSIS AND PROGRESSION

The practicalities of *EGFR* mutation testing, either at diagnosis or at the point of progression, warrant careful consideration, particularly considering recent developments in liquid biopsy techniques. Diagnostic decisions regarding sample type and the timing of the test can have a critical influence on prognostic outcomes, and are discussed below; an author-drafted *EGFR* mutation testing algorithm is also presented in Figure 3.

### Conventional biopsy

Historically, tumor-derived tissue has been instrumental in the elucidation of mechanisms of EGFR TKI action and secondary resistance [110]. Tissue biopsy represents the current gold standard sample type for *EGFR* mutation testing in NSCLC [110] and current guidelines (European Society for Medical Oncology [ESMO], American Society of Clinical Oncology [ASCO], and NCCN) advocate tumor subtype definition as a fundamental step in the diagnostic process [2, 49, 50]. Given its prominence, procedural techniques are highly standardized and widely accessible [111]. However, tissue biopsy has several associated limitations, one of which relates to limited availability of evaluable tissue samples, perhaps due to tumor location or perceived risk to the patient [11, 110, 112]. Such difficulties may also preclude the periodic monitoring of mutation status using sequential tissue samples. Furthermore, whilst there are advances in technologies facilitating the thorough analysis of mutations in archival formalin-fixed paraffin-embedded tissue samples [113] (noting that it is advisable to use strand-specific capture technologies), there are remaining issues associated with the denaturation and fragmentation of DNA [111, 114–116]. Importantly, the usefulness of tissue biopsy techniques may also be confounded by inter-/intra-metastatic tumor heterogeneity, the clinical relevance of which has been discussed previously [33, 110, 115, 117].

### Liquid biopsy

“Liquid biopsies” can provide access to a relative abundance of tumoral genetic material, including circulating free tumor-derived DNA (ctDNA), circulating tumor cells, and exosome vesicles, including exo-DNA [110, 117, 118]. Blood (plasma)-derived ctDNA in particular may represent an option for the identification and monitoring of *EGFR* mutations in patients with NSCLC [117], given the high rates of concordance with matched tumor samples when robust mutation testing methodologies are utilized in stringent research settings [11, 119–121], noting that this is not always the case in clinical practice [122]. Importantly, the presence of *EGFR* mutations in ctDNA has been shown to predict response to EGFR TKIs [23, 119], with similar objective response rates and progression-free survival observed in patients *EGFR* mutation-positive by ctDNA sample versus tissue sample [119, 123].

Compared with conventional tissue biopsy, the liquid biopsy is minimally invasive, allowing for regular, repeated sampling, with a faster turnaround time compared with tissue biopsy [124]. Consequently, this allows for the possibility of early disease detection, along with real-time monitoring of disease progression, treatment response, or evolution of resistance — in some instances months before disease progression is clinically evident [110, 120, 125]. Crucially, liquid biopsy allows for the periodic assessment of tumor heterogeneity [86], provided that the chosen assay can detect somatic mutations, structural variants, and copy number changes. Despite these apparent benefits, the clinical application of ctDNA mutation testing methodologies has yet to be fully realized beyond use in settings with specific, approved, companion diagnostics, and lack of international standardization can limit the accuracy of outcomes [117]. The large, multicenter ASSESS and IGNITE diagnostic studies, which evaluated real-world *EGFR* mutation testing techniques across Europe, Asia, and Russia, observed great variation in testing methodologies, which subsequently impacted the mutation status concordance between ctDNA and matched tissue samples [11, 121]. It is further acknowledged that the robust and sensitive techniques specifically optimized for ctDNA mutation analysis may not be available in all laboratories, which is an important barrier to the adoption of these techniques into routine clinical practice [11, 110, 112]. Other disadvantages include the inability to detect morphologic changes within the tumor (i.e. transformation to another entity as a resistance mechanism) and the potential variability associated with pre-analytical preparation methodologies [126]. Furthermore, the amount of ctDNA available for analysis, when conducted, may not be sufficient to definitively rule out a specific mutation with, amongst other factors, the rate of tumor shedding impacting the fraction of mutant DNA in the bloodstream [126], and repeat testing may be necessary. Most importantly, single-gene diagnostic assays for liquid biopsy (for example, for *EGFR* mutations including T790M) do not provide information on other genetic mechanisms of resistance (e.g. amplification of *MET*). Thus, liquid biopsy molecular multiplex assays, such as next-generation sequencing, are needed to provide information comparable to that obtained from tissue biopsy. Technologies are under development (e.g. hybrid capture assays); however, sensitivity is not currently high enough to substitute tissue-based next-generation sequencing diagnostics.

### Radiomics

Radiomics involves the post-processing and analysis of large amounts of quantitative imaging patient data for diagnostic, predictive, and prognostic modelling. Preliminary data in lung cancer have been reported [127]; however, findings were predominantly based on retrospective analysis. Given the very early stage of development, in depth discussion of radiomics is beyond the scope of this review.

### *EGFR* mutation testing – what, when, and how?

#### What to test?

At diagnosis, the collection of a tumor sample is inevitable for histologic sub-classification, molecular analysis, and treatment choice. At disease progression, the preferred sample type for mutation analysis remains tumor tissue (or cytology) from a progressive lesion, where evaluable. However, ctDNA may be considered as an additional option to tumor tissue at progression, due to its less-invasive nature and the possibility of repeat testing allowing for assessment of disease heterogeneity that arises at progression. However, if a ctDNA sample yields an *EGFR* mutation-negative test result, contradictory to the initial biopsy, a new tissue sample should be obtained to confirm this [126]. This approach is limited, however, by the possibility that alternative mechanisms of resistance may not currently be detected in ctDNA analysis (i.e. protein-based biomarkers or SCLC transformation). In this context, it could be argued that tumor tissue testing is more appropriate at progression. As a consequence, guidelines [50], including the current German S3-Clinical Practice Guidelines (published summer 2017), call for tissue re-biopsy, wherever possible, with liquid biopsy an additional option.

#### When to test?

Mutation testing is advocated at diagnosis to confirm suitability for targeted therapies. As noted previously, progression can be defined in terms of a worsening of tumor burden or the emergence of secondary mutations that confer resistance to the ongoing therapy. Clinical progression is most commonly assessed in accordance with Response Evaluation Criteria in Solid Tumors (RECIST [128]). Whilst currently tumor re-testing is performed on the basis of suspected progression due to radiologic criteria, clinical indicators of progression often lag behind changes seen at the molecular level. Advances in clinical research that allow for the real-time analysis of molecular outcomes and the early detection of biomarkers associated with resistance may reconcile visible symptomatology with changes at the molecular level.

#### How to test?

*EGFR* mutation analysis methodologies for both tissue- and liquid-based testing include laboratory, in-house, and commercial technologies, and are discussed in detail elsewhere [129–131]. Optimal methodologies must be robust, sensitive, and tailored towards the relevant sample type. The sensitivity of the assay is particularly important for ctDNA, given that the amount of ctDNA that is present may be very low and highly fragmented and, consequently, methods used for tissue analysis may not be suitable or require adjustment. Sensitive methods include allele-specific polymerase chain reaction (PCR; e.g. RotorGene Kits [Qiagen], Cobas Kits [Roche], Amoy Kits [Zytomed]), droplet digital PCR (e.g. BioRad), next-generation sequencing, multiplex PCR, or hybridization-based methods (e.g. Qiagen, Illumina, Thermo Fisher, Multiplicom, Agilent) and matrix-assisted laser desorption/ionization time of flight (MALDI-TOF) mass spectrometry. Sanger sequencing or pyrosequencing are not suitable for ctDNA mutation analysis due to low sensitivity.

### Experience with *de novo* T790M mutations (allele frequency tested in parallel)

Baseline T790M mutations have been detected in EGFR TKI-naïve patients, and in brain metastases, at low rates (<1%) using standard molecular analysis [31, 132]. Data obtained using more sensitive methodology (e.g. MALDI-TOF mass spectrometry, TaqMan quantitative PCR, amplification refractory mutation system) suggest that the prevalence of *de novo* mutations may be much higher (22–25%) [132, 133], although replication in larger patient samples is warranted before firm conclusions can be drawn. Regardless of prevalence, given that approved, first-line EGFR TKI therapies have proven to be of limited value in these patients [31, 134], the possibility of such mutations should be considered in any diagnostic approach.

## RESISTANCE TO “THIRD-GENERATION” TKIS

### C797S as a mechanism of TKI resistance

As previously discussed, “third-generation” EGFR TKIs target the T790M mutation [63, 66, 135]. However, recent clinical findings have suggested the emergence of a tertiary acquired *EGFR* mutation *C797S* after treatment with a “third-generation” TKI [27, 30, 31, 80]. “Third-generation” TKIs rely in part on the formation of a covalent bond between the TKI and the 797-cysteine residue for their strong binding to the EGFR, but the mutation of the 797-cysteine residue to a serine (*C797S*) prevents such bond formation, compromising the TKI’s efficacy and leading to subsequent resistance [96]. Combination strategies of first- and third-generation EGFR TKIs have been used to target C797S resistance to third-generation EGFR TKIs [136, 137]. Evidence supporting this approach comes from *in vitro* studies, which have shown that presence of T790M and C797S in trans (on different DNA strands), leads to resistance to third-generation EGFR TKI but a sensitivity to a combination therapy of first- and third-generation EGFR TKIs [138]. Recently, two case reports have shown that this combination EGFR TKI approach is effective in patients harbouring the T790M and C797S mutation in trans. However, these mutations can also occur in cis (on the same DNA strand), mediating treatment resistance prior to, or during, combination therapy. Thus, determining if the mutations are cis- or trans-allelic could be used to predict the outcome of this combinatorial therapy [136, 137].

### Other mechanisms of resistance

It must be noted that patterns of resistance to “third-generation” TKIs may differ between settings, e.g. with or without the presence of a T790M mutation, and that the emergence of resistance to “third-generation” TKIs is not limited to the *EGFR* C797S mutation. It can occur via multiple mechanisms, including, but not limited to: MAPK1 amplification, downregulation of negative regulators of ERK, NRAS mutation/amplification, and KRAS amplification [27]. Resistance to rociletinib and osimertinib, whilst not yet fully understood, is thought to recurrently involve MET, EGFR, PIK3CA, ERRB2, KRAS, and RB1 pathways, as well as the possibility of neuroendocrine transformation to SCLC [27, 86, 108].

### Rationale for combination therapies

Given the prevalence of multiple resistance mechanisms, a single therapeutic agent may not be sufficient to treat a genetically heterogeneous and rapidly evolving tumor [35]. This has prompted combinatorial approaches to drug management that are tailored to the heterogeneity of the specific tumor [33] and which broadly fall into two categories: first-line combination therapies that delay the emergence of resistance, and later-line combinations for use after progression has occurred that directly target resistance. Treatment combinations currently under pre-clinical and clinical investigation are summarized in Table 2. Preliminary data appear promising but may be compromised by increased risk of toxicity [139].

## FUTURE OUTLOOK

While the nature of acquired resistance mechanisms in NSCLC continues to evolve, we look to the future application of advances in our understanding in this area, in terms of clinical decision-making. Post-progression treatment should be tailored according to identified resistance mechanisms and clinical characteristics.

The identification of primary *EGFR*-sensitizing mutations at screening guides initial treatment approaches and is well established. However, the continued elucidation of the evolutionary mechanisms of acquired resistance in line with changes in individual tumor heterogeneity means that molecular targets are continuously changing, and therapeutic approaches must adapt accordingly. Whilst this remains a significant clinical challenge, advances in molecular profiling techniques, particularly the advent of liquid biopsy, may ultimately make possible real-time, holistic analysis of tumor behavior, thereby further informing appropriate treatment decisions.

As the range of molecularly targeted therapies broadens, it will be increasingly feasible to tailor treatment to the individual patient in response to changes in their genomic profile. Where possible, the immediate incorporation of emerging scientific approaches into current clinical practice will improve outcomes for patients with advanced NSCLC.

## References

[1] American Cancer Society (2016). Cancer Facts & Figures.

[2] Novello S, Barlesi F, Califano R, Cufer T, Ekman S, Levra MG, Kerr K, Popat S, Reck M, Senan S, Simo GV, Vansteenkiste J, Peters S (2016). Metastatic non-small-cell lung cancer: ESMO clinical practice guidelines for diagnosis, treatment and follow-up. Ann Oncol.

[3] Gadgeel SM (2016). Personalized therapy of non-small cell lung cancer (NSCLC). Adv Exp Med Biol.

[4] Leighl NB (2012). Treatment paradigms for patients with metastatic non-small-cell lung cancer: first-, second-, and third-line. Curr Oncol.

[5] Clinical Lung Cancer Genome Project (CLCGP), and Network Genomic Medicine (NGM) (2013). A genomics-based classification of human lung tumors. Sci Transl Med.

[6] Dearden S, Stevens J, Wu YL, Blowers D (2013). Mutation incidence and coincidence in non small-cell lung cancer: meta-analyses by ethnicity and histology (mutMap). Ann Oncol.

[7] Planchard D, Smit EF, Groen HJ, Mazieres J, Besse B, Å Helland, Giannone V, D’Amelio AM, Zhang P, Mookerjee B, Johnson BE (2017). Dabrafenib plus trametinib in patients with previously untreated BRAFV600E-mutant metastatic non-small-cell lung cancer: an open-label, phase 2 trial. Lancet Oncol.

[8] Barlesi F, Mazieres J, Merlio JP, Debieuvre D, Mosser J, Lena H, Ouafik L, Besse B, Rouquette I, Westeel V, Escande F, Monnet I, Lemoine A, Biomarkers France Contributors (2016). Routine molecular profiling of patients with advanced non-small-cell lung cancer: results of a 1-year nationwide programme of the French Cooperative Thoracic Intergroup (IFCT). Lancet.

[9] Kim ES, Hirsh V, Mok T, Socinski MA, Gervais R, Wu YL, Li LY, Watkins CL, Sellers MV, Lowe ES, Sun Y, Liao ML, Østerlind K (2008). Gefitinib versus docetaxel in previously treated non-small-cell lung cancer (INTEREST): a randomised phase III trial. Lancet.

[10] Kris MG, Johnson BE, Berry LD, Kwiatkowski DJ, Iafrate AJ, Wistuba II, Varella-Garcia M, Franklin WA, Aronson SL, Su PF, Shyr Y, Camidge DR, Sequist LV (2014). Using multiplexed assays of oncogenic drivers in lung cancers to select targeted drugs. JAMA.

[11] Reck M, Hagiwara K, Han B, Tjulandin S, Grohé C, Yokoi T, Morabito A, Novello S, Arriola E, Molinier O, McCormack R, Ratcliffe M, Normanno N (2016). ctDNA determination of EGFR mutation status in European and Japanese patients with advanced NSCLC: the ASSESS study. J Thorac Oncol.

[12] Schuette W, Schirmacher P, Eberhardt WE, Fischer JR, von der Schulenburg JM, Mezger J, Schumann C, Serke M, Zaun S, Dietel M, Thomas M (2015). EGFR mutation status and first-line treatment in patients with stage III/IV non-small cell lung cancer in Germany: an observational study. Cancer Epidemiol Biomarkers Prev.

[13] Shepherd FA, Rodrigues Pereira J, Ciuleanu T, Tan EH, Hirsh V, Thongprasert S, Campos D, Maoleekoonpiroj S, Smylie M, Martins R, van Kooten M, Dediu M, Findlay B, National Cancer Institute of Canada Clinical Trials Group (2005). Erlotinib in previously treated non-small-cell lung cancer. N Engl J Med.

[14] Shi Y, Au JS, Thongprasert S, Srinivasan S, Tsai CM, Khoa MT, Heeroma K, Itoh Y, Cornelio G, Yang PC (2014). A prospective, molecular epidemiology study of EGFR mutations in Asian patients with advanced non-small-cell lung cancer of adenocarcinoma histology (PIONEER). J Thorac Oncol.

[15] Thatcher N, Chang A, Parikh P, Rodrigues PJ, Ciuleanu T, von Pawel J, Thongprasert S, Tan EH, Pemberton K, Archer V, Carroll K (2005). Gefitinib plus best supportive care in previously treated patients with refractory advanced non-small-cell lung cancer: results from a randomised, placebo-controlled, multicentre study (Iressa Survival Evaluation in Lung Cancer). Lancet.

[16] Chan BA, Hughes BG (2015). Targeted therapy for non-small cell lung cancer: current standards and the promise of the future. Transl Lung Cancer Res.

[17] Lin JJ, Cardarella S, Lydon CA, Dahlberg SE, Jackman DM, Jänne PA, Johnson BE (2016). Five-year survival in EGFR-mutant metastatic lung adenocarcinoma treated with EGFR-TKIs. J Thorac Oncol.

[18] Greenhalgh J, Dwan K, Boland A, Bates V, Vecchio F, Dundar Y, Jain P, Green JA (2016). First-line treatment of advanced epidermal growth factor receptor (EGFR) mutation positive non-squamous non-small cell lung cancer. Cochrane Database Syst Rev.

[19] Fukuoka M, Wu YL, Thongprasert S, Sunpaweravong P, Leong SS, Sriuranpong V, Chao TY, Nakagawa K, Chu DT, Saijo N, Duffield EL, Rukazenkov Y, Speake G (2011). Biomarker analyses and final overall survival results from a Phase III, randomized, open-label, first-line study of gefitinib versus carboplatin/paclitaxel in clinically selected patients with advanced non-small-cell lung cancer in Asia (IPASS). J Clin Oncol.

[20] Maemondo M, Inoue A, Kobayashi K, Sugawara S, Oizumi S, Isobe H, Gemma A, Harada M, Yoshizawa H, Kinoshita I, Fujita Y, Okinaga S, Hirano H, North-East Japan Study Group (2010). Gefitinib or chemotherapy for non-small-cell lung cancer with mutated EGFR. N Engl J Med.

[21] Mitsudomi T, Morita S, Yatabe Y, Negoro S, Okamoto I, Tsurutani J, Seto T, Satouchi M, Tada H, Hirashima T, Asami K, Katakami N, Takada M, West Japan Oncology Group (2010). Gefitinib versus cisplatin plus docetaxel in patients with non-small-cell lung cancer harbouring mutations of the epidermal growth factor receptor (WJTOG3405): an open label, randomised phase 3 trial. Lancet Oncol.

[22] Mok TS, Wu YL, Thongprasert S, Yang CH, Chu DT, Saijo N, Sunpaweravong P, Han B, Margono B, Ichinose Y, Nishiwaki Y, Ohe Y, Yang JJ (2009). Gefitinib or carboplatin-paclitaxel in pulmonary adenocarcinoma. N Engl J Med.

[23] Rosell R, Carcereny E, Gervais R, Vergnenegre A, Massuti B, Felip E, Palmero R, Garcia-Gomez R, Pallares C, Sanchez JM, Porta R, Cobo M, Garrido P, Spanish Lung Cancer Group in collaboration with Groupe Français de Pneumo-Cancérologie and Associazione Italiana Oncologia Toracica (2012). Erlotinib versus standard chemotherapy as first-line treatment for European patients with advanced EGFR mutation-positive non-small-cell lung cancer (EURTAC): a multicentre, open-label, randomised phase 3 trial. Lancet Oncol.

[24] Sequist LV, Yang JC, Yamamoto N, O’Byrne K, Hirsh V, Mok T, Geater SL, Orlov S, Tsai CM, Boyer M, Su WC, Bennouna J, Kato T (2013). Phase III study of afatinib or cisplatin plus pemetrexed in patients with metastatic lung adenocarcinoma with EGFR mutations. J Clin Oncol.

[25] Yang JC, Wu YL, Schuler M, Sebastian M, Popat S, Yamamoto N, Zhou C, Hu CP, O’Byrne K, Feng J, Lu S, Huang Y, Geater SL (2015). Afatinib versus cisplatin-based chemotherapy for EGFR mutation-positive lung adenocarcinoma (LUX-Lung 3 and LUX-Lung 6): analysis of overall survival data from two randomised, phase 3 trials. Lancet Oncol.

[26] Zhou C, Wu YL, Chen G, Feng J, Liu XQ, Wang C, Zhang S, Wang J, Zhou S, Ren S, Lu S, Zhang L, Hu C (2011). Erlotinib versus chemotherapy as first-line treatment for patients with advanced EGFR mutation-positive non-small-cell lung cancer (OPTIMAL, CTONG-0802): a multicentre, open-label, randomised, phase 3 study. Lancet Oncol.

[27] Costa DB, Kobayashi SS (2015). Whacking a mole-cule: clinical activity and mechanisms of resistance to third generation EGFR inhibitors in EGFR mutated lung cancers with EGFR-T790M. Transl Lung Cancer Res.

[28] Ercan D, Choi HG, Yun CH, Capelletti M, Xie T, Eck MJ, Gray NS, Janne PA (2015). EGFR mutations and resistance to irreversible pyrimidine-based EGFR inhibitors. Clin Cancer Res.

[29] Niederst MJ, Hu H, Mulvey HE, Lockerman EL, Garcia AR, Piotrowska Z, Sequist LV, Engelman JA (2015). The allelic context of the C797S mutation acquired upon treatment with third-generation EGFR inhibitors impacts sensitivity to subsequent treatment strategies. Clin Cancer Res.

[30] Thress KS, Paweletz CP, Felip E, Cho BC, Stetson D, Dougherty B, Lai Z, Markovets A, Vivancos A, Kuang Y, Ercan D, Matthews SE, Cantarini M (2015). Acquired EGFR C797S mutation mediates resistance to AZD9291 in non-small cell lung cancer harboring EGFR T790M. Nat Med.

[31] Yu HA, Tian SK, Drilon AE, Borsu L, Riely GJ, Arcila ME, Ladanyi M (2015). Acquired resistance of EGFR-mutant lung cancer to a T790M-specific EGFR inhibitor: emergence of a third mutation (C797S) in the EGFR tyrosine kinase domain. JAMA Oncol.

[32] Burrell RA, McGranahan N, Bartek J, Swanton C (2013). The causes and consequences of genetic heterogeneity in cancer evolution. Nature.

[33] Fisher R, Pusztai L, Swanton C (2013). Cancer heterogeneity: implications for targeted therapeutics. Br J Cancer.

[34] Jamal-Hanjani M, Quezada SA, Larkin J, Swanton C (2015). Translational implications of tumor heterogeneity. Clin Cancer Res.

[35] Scheffler M, Merkelbach-Bruse S, Bos M, Fassunke J, Gardizi M, Michels S, Groneck L, Schultheis AM, Malchers F, Leenders F, Kobe C, König K, Heukamp LC (2015). Spatial tumor heterogeneity in lung cancer with acquired epidermal growth factor receptor-tyrosine kinase inhibitor resistance: targeting high-level MET-amplification and EGFR T790M mutation occurring at different sites in the same patient. J Thorac Oncol.

[36] Furugen M, Uechi K, Hirai J, Aoyama H, Saio M, Yoshimi N, Kinjo T, Miyagi K, Haranaga S, Higa F, Tateyama M, Fujita J (2015). An Autopsy Case of Two Distinct, Acquired Drug Resistance Mechanisms in Epidermal Growth Factor Receptor-mutant Lung Adenocarcinoma: Small Cell Carcinoma Transformation and Epidermal Growth Factor Receptor T790M Mutation. Intern Med.

[37] Belchis DA, Tseng LH, Gniadek T, Haley L, Lokhandwala P, Illei P, Gocke CD, Forde P, Brahmer J, Askin FB, Eshleman JR, Lin MT (2016). Heterogeneity of resistance mutations detectable by nextgeneration sequencing in TKI-treated lung adenocarcinoma. Oncotarget.

[38] Zhang J, Fujimoto J, Zhang J, Wedge DC, Song X, Zhang J, Seth S, Chow CW, Cao Y, Gumbs C, Gold KA, Kalhor N, Little L (2014). Intratumor heterogeneity in localized lung adenocarcinomas delineated by multiregion sequencing. Science.

[39] Chen Z, Fillmore CM, Hammerman PS, Kim CF, Wong KK (2014). Non-small-cell lung cancers: a heterogeneous set of diseases. Nat Rev Cancer.

[40] Engelman JA, Settleman J (2008). Acquired resistance to tyrosine kinase inhibitors during cancer therapy. Curr Opin Genet Dev.

[41] Gerlinger M, Swanton C (2010). How Darwinian models inform therapeutic failure initiated by clonal heterogeneity in cancer medicine. Br J Cancer.

[42] Misale S, Yaeger R, Hobor S, Scala E, Janakiraman M, Liska D, Valtorta E, Schiavo R, Buscarino M, Siravegna G, Bencardino K, Cercek A, Chen CT (2012). Emergence of KRAS mutations and acquired resistance to anti-EGFR therapy in colorectal cancer. Nature.

[43] Maheswaran S, Sequist LV, Nagrath S, Ulkus L, Brannigan B, Collura CV, Inserra E, Diederichs S, Iafrate AJ, Bell DW, Digumarthy S, Muzikansky A, Irimia D (2008). Detection of mutations in EGFR in circulating lung-cancer cells. N Engl J Med.

[44] Zellmer VR, Zhang S (2014). Evolving concepts of tumor heterogeneity. Cell Biosci.

[45] Cooper WA, Lam DC, O’Toole SA, Minna JD (2013). Molecular biology of lung cancer. J Thorac Dis.

[46] Sholl LM, Aisner DL, Varella-Garcia M, Berry LD, Dias-Santagata D, Wistuba II, Chen H, Fujimoto J, Kugler K, Franklin WA, Iafrate AJ, Ladanyi M, Kris MG, Investigators. LCMC (2015). Multi-institutional oncogenic driver mutation analysis in lung adenocarcinoma: the Lung Cancer Mutation Consortium experience. J Thorac Oncol.

[47] Goeckenjan G, Sitter H, Thomas M, Branscheid D, Flentje M, Griesinger F, Niederle N, Stuschke M, Blum T, Deppermann KM, Ficker JH, Freitag L, Lübbe AS, Deutsche Gesellschaft für Pneumologie und Beatmungsmedizin und die Deutsche Krebsgesellschaft (2010). [Prevention, diagnosis, therapy, and follow-up of lung cancer]. [Article in German]. Pneumologie.

[48] Leighl NB, Rekhtman N, Biermann WA, Huang J, Mino-Kenudson M, Ramalingam SS, West H, Whitlock S, Somerfield MR (2014). Molecular testing for selection of patients with lung cancer for epidermal growth factor receptor and anaplastic lymphoma kinase tyrosine kinase inhibitors: American Society of Clinical Oncology endorsement of the College of American Pathologists/International Association for the study of lung cancer/association for molecular pathology guideline. J Clin Oncol.

[49] Masters GA, Temin S, Azzoli CG, Giaccone G, Baker S, Brahmer JR, Ellis PM, Gajra A, Rackear N, Schiller JH, Smith TJ, Strawn JR, Trent D, Johnson DH, American Society of Clinical Oncology Clinical Practice (2015). Systemic therapy for stage IV non-small-cell lung cancer: American Society of Clinical Oncology clinical practice guideline update. J Clin Oncol.

[50] National Comprehensive Cancer Network Practice guidelines in oncology – version V.4.2017 (non-small-cell lung cancer). https://www.nccn.org/store/login/login.aspx?ReturnURL=https://www.nccn.org/professionals/physician_gls/pdf/nscl.pdf.

[51] Piotrowska Z, Sequist LV (2016). Treatment of EGFR-mutant lung cancers after progression in patients receiving first-line EGFR tyrosine kinase inhibitors: a review. JAMA Oncol.

[52] Yap TA, Macklin-Doherty A, Popat S (2017). Continuing EGFR inhibition beyond progression in advanced non-small cell lung cancer. Eur J Cancer.

[53] Weickhardt AJ, Scheier B, Burke JM, Gan G, Lu X, Bunn PA, Aisner DL, Gaspar LE, Kavanagh BD, Doebele RC, Camidge DR (2012). Local ablative therapy of oligoprogressive disease prolongs disease control by tyrosine kinase inhibitors in oncogene-addicted non-small-cell lung cancer. J Thorac Oncol.

[54] Yu HA, Sima CS, Huang J, Solomon SB, Rimner A, Paik P, Pietanza MC, Azzoli CG, Rizvi NA, Krug LM, Miller VA, Kris MG, Riely GJ (2013). Local therapy with continued EGFR tyrosine kinase inhibitor therapy as a treatment strategy in EGFR-mutant advanced lung cancers that have developed acquired resistance to EGFR tyrosine kinase inhibitors. J Thorac Oncol.

[55] Park K, Yu CJ, Kim SW, Lin MC, Sriuranpong V, Tsai CM, Lee JS, Kang JH, Chan KC, Perez-Moreno P, Button P, Ahn MJ, Mok T (2016). First-line erlotinib therapy until and beyond Response Evaluation Criteria in Solid Tumors progression in Asian patients with epidermal growth factor receptor mutation-positive non-small-cell lung cancer: the ASPIRATION study. JAMA Oncol.

[56] Yu HA, Arcila ME, Rekhtman N, Sima CS, Zakowski MF, Pao W, Kris MG, Miller VA, Ladanyi M, Riely GJ (2013). Analysis of tumor specimens at the time of acquired resistance to EGFR-TKI therapy in 155 patients with EGFR-mutant lung cancers. Clin Cancer Res.

[57] Kwak EL, Sordella R, Bell DW, Godin-Heymann N, Okimoto RA, Brannigan BW, Harris PL, Driscoll DR, Fidias P, Lynch TJ, Rabindran SK, McGinnis JP, Wissner A (2005). Irreversible inhibitors of the EGF receptor may circumvent acquired resistance to gefitinib. Proc Natl Acad Sci U S A.

[58] Pao W, Miller VA, Politi KA, Riely GJ, Somwar R, Zakowski MF, Kris MG, Varmus H (2005). Acquired resistance of lung adenocarcinomas to gefitinib or erlotinib is associated with a second mutation in the EGFR kinase domain. PLoS Med.

[59] Sequist LV, Waltman BA, Dias-Santagata D, Digumarthy S, Turke AB, Fidias P, Bergethon K, Shaw AT, Gettinger S, Cosper AK, Akhavanfard S, Heist RS, Temel J (2011). Genotypic and histological evolution of lung cancers acquiring resistance to EGFR inhibitors. Sci Transl Med.

[60] Azam M, Seeliger MA, Gray NS, Kuriyan J, Daley GQ (2008). Activation of tyrosine kinases by mutation of the gatekeeper threonine. Nat Struct Mol Biol.

[61] Yun CH, Mengwasser KE, Toms AV, Woo MS, Greulich H, Wong KK, Meyerson M, Eck MJ (2008). The T790M mutation in EGFR kinase causes drug resistance by increasing the affinity for ATP. Proc Natl Acad Sci U S A.

[62] Kobayashi S, Boggon TJ, Dayaram T, Janne PA, Kocher O, Meyerson M, Johnson BE, Eck MJ, Tenen DG, Halmos B (2005). EGFR mutation and resistance of non-small-cell lung cancer to gefitinib. N Engl J Med.

[63] Jänne PA, Yang JC, Kim DW, Planchard D, Ohe Y, Ramalingam SS, Ahn MJ, Kim SW, Su WC, Horn L, Haggstrom D, Felip E, Kim JH (2015). AZD9291 in EGFR inhibitor-resistant non-small-cell lung cancer. N Engl J Med.

[64] Goss G, Tsai CM, Shepherd FA, Bazhenova L, Lee JS, Chang GC, Crino L, Satouchi M, Chu Q, Hida T, Han JY, Juan O, Dunphy F (2016). Osimertinib for pretreated EGFR Thr790Met-positive advanced non-small-cell lung cancer (AURA2): a multicentre, open-label, single-arm, phase 2 study. Lancet Oncol.

[65] Mok TS, Wu YL, Ahn MJ, G arassino MC, Kim HR, Ramalingam SS, Shepherd FA, He Y, Akamatsu H, Theelen WS, Lee CK, Sebastian M, Templeton A, AURA3 Investigators (2017). Osimertinib or platinum-pemetrexed in EGFR T790M-positive lung cancer. N Engl J Med.

[66] Sequist LV, Soria JC, Goldman JW, Wakelee HA, Gadgeel SM, Varga A, Papadimitrakopoulou V, Solomon BJ, Oxnard GR, Dziadziuszko R, Aisner DL, Doebele RC, Galasso C (2015). Rociletinib in EGFR-mutated non-small-cell lung cancer. N Engl J Med.

[67] Clovis Oncology Inc Clovis Oncology announces regulatory update for rociletinib NDA filing. http://www.businesswire.com/news/home/20151116005513/en/.

[68] Sequist LV, Soria JC, Camidge DR (2016). Update to rociletinib data with the RECIST confirmed response rate. N Engl J Med.

[69] Park K, Lee JS, Lee KH, Kim JH, Min YJ, Cho JY, Han JY, Kim BS, Kim JS, Lee DH, Kang JH, Cho EK, Jang IJ (2015). Updated safety and efficacy results from phase I/II study of HM61713 in patients (pts) with EGFR mutation positive non-small cell lung cancer (NSCLC) who failed previous EGFR-tyrosine kinase inhibitor (TKI). J Clin Oncol.

[70] Tan DS, Seto T, Leighl NB, Riely GJ, Sequist LV, Felip E, Wolf J, Yang JC, Matushansky I, Yu X, Schmitz SF, Cui X, Kim DW (2015). First-in-human phase I study of EGF816, a third generation, mutant-selective EGFR tyrosine kinase inhibitor, in advanced n on-small cell lung cancer (NSCLC) harboring T790M. J Clin Oncol.

[71] Jia Y, Juarez J, Li J, Manuia M, Niederst MJ, Tompkins C, Timple N, Vaillancourt MT, Pferdekamper AC, Lockerman EL, Li C, Anderson J, Costa C (2016). EGF816 exerts anticancer effects in non-small cell lung cancer by irreversibly and selectively targeting primary and acquired activating mutations in the EGF receptor. Cancer Res.

[72] Yu HA, Spira AI, Horn L, Weis J, West HJ, Giaccone G, Evans TL, Kelly RJ, Desai BB, Krivoshik A, Fleege TE, Poondru S, Jie F (2016). Antitumor activity of ASP8273 300 mg in subjects with EGFR mutation-positive non-small cell lung cancer: interim results from an ongoing phase 1 study. J Clin Oncol.

[73] Wang S, Cang S, Liu D (2016). Third-generation inhibitors targeting EGFR T790M mutation in advanced non-small cell lung cancer. J Hematol Oncol.

[74] Schildhaus HU, Schultheis AM, Rüschoff J, Binot E, Merkelbach-Bruse S, Fassunke J, Schulte W, Ko YD, Schlesinger A, Bos M, Gardizi M, Engel-Riedel W, Brockmann M (2015). MET amplification status in therapy-naïve adeno- and squamous cell carcinomas of the lung. Clin Cancer Res.

[75] Arcila ME, Oxnard GR, Nafa K, Riely GJ, Solomon SB, Zakowski MF, Kris MG, Pao W, Miller VA, Ladanyi M (2011). Rebiopsy of lung cancer patients with acquired resistance to EGFR inhibitors and enhanced detection of the T790M mutation using a locked nucleic acid-based assay. Clin Cancer Res.

[76] Bean J, Brennan C, Shih JY, Riely G, Viale A, Wang L, Chitale D, Motoi N, Szoke J, Broderick S, Balak M, Chang WC, Yu CJ (2007). MET amplification occurs with or without T790M mutations in EGFR mutant lung tumors with acquired resistance to gefitinib or erlotinib. Proc Natl Acad Sci U S A.

[77] Chen HJ, Mok TS, Chen ZH, Guo AL, Zhang XC, Su J, Wu YL (2009). Clinicopathologic and molecular features of epidermal growth factor receptor T790M mutation and c-MET amplification in tyrosine kinase inhibitor-resistant Chinese non-small cell lung cancer. Pathol Oncol Res.

[78] Cortot AB, Jänne PA (2014). Molecular mechanisms of resistance in epidermal growth factor receptor-mutant lung adenocarcinomas. Eur Respir Rev.

[79] Engelman JA, Zejnullahu K, Mitsudomi T, Song Y, Hyland C, Park JO, Lindeman N, Gale CM, Zhao X, Christensen J, Kosaka T, Holmes AJ, Rogers AM (2007). MET amplification leads to gefitinib resistance in lung cancer by activating ERBB3 signaling. Science.

[80] Ortiz-Cuaran S, Scheffler M, Plenker D, Dahmen L, Scheel AH, Fernandez-Cuesta L, Meder L, Lovly CM, Persigehl T, Merkelbach-Bruse S, Bos M, Michels S, Fischer R (2016). Heterogeneous mechanisms of primary and acquired resistance to third-generation EGFR inhibitors. Clin Cancer Res.

[81] Pérez-Ramírez C, Cañadas-Garre M, Jiménez-Varo E, Faus-Dáder MJ, Calleja-Hernández MÁ (2015). MET: a new promising biomarker in non-small-cell lung carcinoma. Pharmacogenomics.

[82] Turke AB, Zejnullahu K, Wu YL, Song Y, Dias-Santagata D, Lifshits E, Toschi L, Rogers A, Mok T, Sequist L, Lindeman NI, Murphy C, Akhavanfard S (2010). Preexistence and clonal selection of MET amplification in EGFR mutant NSCLC. Cancer Cell.

[83] Gou LY, Li AN, Yang JJ, Zhang XC, Su J, Yan HH, Xie Z, Lou NN, Liu SY, Dong ZY, Gao HF, Zhou Q, Zhong WZ (2016). The c oexistence of MET over-expression and an EGFR T790M mutation is related to acquired resistance to EGFR tyrosine kinase inhibitors in advanced non-small cell lung cancer. Oncotarget.

[84] Ma C, Wei S, Song Y (2011). T790M and acquired resistance of EGFR TKI: a literature review of clinical reports. J Thorac Dis.

[85] Xu L, Kikuchi E, Xu C, Ebi H, Ercan D, Cheng KA, Padera R, Engelman JA, Jãnne PA, Shapiro GI, Shimamura T, Wong KK (2012). Combined EGFR/MET or EGFR/HSP90 inhibition is effective in the treatment of lung cancers codriven by mutant EGFR containing T790M and MET. Cancer Res.

[86] Chabon JJ, Simmons AD, Lovejoy AF, Esfahani MS, Newman AM, Haringsma HJ, Kurtz DM, Stehr H, Scherer F, Karlovich CA, Harding TC, Durkin KA, Otterson GA (2016). Circulating tumour DNA profiling reveals heterogeneity of EGFR inhibitor resistance mechanisms in lung cancer patients. Nat Commun.

[87] Nakagawa T, Takeuchi S, Yamada T, Nanjo S, Ishikawa D, Sano T, Kita K, Nakamura T, Matsumoto K, Suda K, Mitsudomi T, Sekido Y, Uenaka T, Yano S (2012). Combined therapy with mutant-selective EGFR inhibitor and Met kinase inhibitor for overcoming erlotinib resistance in EGFR-mutant lung cancer. Mol Cancer Ther.

[88] Nanjo S, Yamada T, Nishihara H, Takeuchi S, Sano T, Nakagawa T, Ishikawa D, Zhao L, Ebi H, Yasumoto K, Matsumoto K, Yano S (2013). Ability of the Met kinase inhibitor crizotinib and new generation EGFR inhibitors to overcome resistance to EGFR inhibitors. PLoS One.

[89] Gibbons DL, Chow LQ, Kim DW, Kim SW, Yeh T, Song X, Jiang H, Taylor R, Karakunnel J, Creelan B (2016). 57O Efficacy, safety and tolerability of MEDI4736 (durvalumab [D]), a human IgG1 anti-programmed cell death-ligand-1 (PD-L1) antibody, combined with gefitinib (G): a phase I expansion in TKI-naive patients (pts) with EGFR mutant NSCLC. J Thorac Oncol.

[90] Oxnard GR, Ramalingam SS, Ahn MJ, Kim SW, Yu HA, Saka H, Horn L, Goto K, Ohe Y, Cantarini M, Frewer P, Lahn M, Yang JC (2015). Preliminary results of TATTON, a multi-arm phase Ib trial of AZD9291 combined with MEDI4736, AZD6094 or selumetinib in EGFR-mutant lung cancer. J Clin Oncol.

[91] Ahn MJ, Yang J, Yu H, Saka H, Ramalingam S, Goto K, Kim SW, Yang L, Walding A, Oxnard GR (2016). 136O: Osimertinib combined with durvalumab in EGFR-mutant non-small cell lung cancer: results from the TATTON phase Ib trial. J Thorac Oncol.

[92] Yang JJ, Yang L, Farnsworth A, Handzel A, Coleman T, Morgan S, Wu YL (2016). Preliminary results of a phase Ib trial of savolitinib com bined with gefitinib in EGFR-mutant lung cancer. J Clin Oncol.

[93] Janjigian YY, Smit EF, Groen HJ, Horn L, Gettinger S, Camidge DR, Riely GJ, Wang B, Fu Y, Chand VK, Miller VA, Pao W (2014). Dual inhibition of EGFR with afatinib and cetuximab in kinase inhibitor-resistant EGFR-mutant lung cancer with and without T790M mutations. Cancer Discov.

[94] Ou SH, Agarwal N, Ali SM (2016). High MET amplification level as a resistance mechanism to osimertinib (AZD9291) in a patient that symptomatically responded to crizotinib treatment post-osimertinib progression. Lung Cancer.

[95] Smit E, Kopp HG, Kim DW, Tortora G, Spira AI, Berruti A, Lee DH, Reguart N, Rybkin II, Akimov M, Schumacher M, Upalawanna A, Xu C (2016). GEOMETRY duo-1: A phase (Ph) Ib/II, multicenter trial of oral cMET inhibitor capmatinib (INC280) ± erlotinib vs platinum + pemetrexed in adult patients (pts) with epidermal growth factor receptor (EGFR)-mutated, cMET-amplified, locally advanced/metastatic non-small cell lung cancer (NSCLC) with acquired resistance to prior EGFR tyrosine kinase inhibitor (TKI) therapy. J Clin Oncol.

[96] Jia Y, Yun CH, Park E, Ercan D, Manuia M, Juarez J, Xu C, Rhee K, Chen T, Zhang H, Palakurthi S, Jang J, Lelais G (2016). Overcoming EGFR(T790M) and EGFR(C797S) resistance with mutant-selective allosteric inhibitors. Nature.

[97] Minari R, Bordi P, Tiseo M (2016). Third-generation epidermal growth factor receptor-tyrosine kinase inhibitors in T790M-positive non-small cell lung cancer: review on emerged mechanisms of resistance. Transl Lung Cancer Res.

[98] Shi P, Oh YT, Zhang G, Yao W, Yue P, Li Y, Kanteti R, Riehm J, Salgia R, Owonikoko TK, Ramalingam SS, Chen M, Sun SY (2016). Met gene amplification and protein hyperactivation is a mechanism of resistance to both first and third generation EGFR inhibitors in lung cancer treatment. Cancer Lett.

[99] Takezawa K, Pirazzoli V, Arcila ME, Nebhan CA, Song X, de Stanchina E, Ohashi K, Janjigian YY, Spitzler PJ, Melnick MA, Riely GJ, Kris MG, Miller VA (2012). HER2 amplification: a potential mechanism of acquired resistance to EGFR inhibition in EGFR-mutant lung cancers that lack the second-site EGFRT790M mutation. Cancer Discov.

[100] Planchard D, Loriot Y, André F, Gobert A, Auger N, Lacroix L, Soria JC (2015). EGFR-independent mechanisms of acquired resistance to AZD9291 in EGFR T790M-positive NSCLC patients. Ann Oncol.

[101] Ross HJ, Blumenschein GR, Aisner J, Damjanov N, Dowlati A, Garst J, Rigas JR, Smylie M, Hassani H, Allen KE, Leopold L, Zaks TZ, Shepherd FA (2010). Randomized phase II multicenter trial of two schedules of lapatinib as first- or second-line monotherapy in patients with advanced or metastatic non-small cell lung cancer. Clin Cancer Res.

[102] Gatzemeier U, Groth G, Butts C, van Zandwijk N, Shepherd F, Ardizzoni A, Barton C, Ghahramani P, Hirsh V (2004). Randomized phase II trial of gemcitabine-cisplatin with or without trastuzumab in HER2-positive non-small-cell lung cancer. Ann Oncol.

[103] Rothschild SI (2015). Targeted therapies in non-small cell lung cancer-beyond EGFR and ALK. Cancers (Basel).

[104] Tricker EM, Xu C, Uddin S, Capelletti M, Ercan D, Ogino A, Pratilas CA, Rosen N, Gray NS, Wong KK, Jänne PA (2015). Combined EGFR/MEK inhibition prevents the emergence of resistance in EGFR-mutant lung cancer. Cancer Discov.

[105] Socinski MA, Villaruz LC, Ross J (2017). Understanding mechanisms of resistance in the epithelial growth factor receptor in non-small cell lung cancer and the role of biopsy at progression. Oncologist.

[106] Wang J, Wang B, Chu H, Yao Y (2016). Intrinsic resistance to EGFR tyrosine kinase inhibitors in advanced non-small-cell lung cancer with activating EGFR mutations. Onco Targets Ther.

[107] Bidkhori G, Moeini A, Masoudi-Nejad A (2012). Modeling of tumor progression in NSCLC and intrinsic resistance to TKI in loss of PTEN expression. PLoS One.

[108] Ham JS, Kim S, Kim HK, Byeon S, Sun JM, Lee SH, Ahn JS, Park K, Choi YL, Han J, Park W, Ahn MJ (2016). Two cases of small cell lung cancer transformation from EGFR mutant adenocarcinoma during AZD9291 treatment. J Thorac Oncol.

[109] Suda K, Murakami I, Sakai K, Mizuuchi H, Shimizu S, Sato K, Tomizawa K, Tomida S, Yatabe Y, Nishio K, Mitsudomi T (2015). Small cell lung cancer transformation and T790M mutation: complimentary roles in acquired resistance to kinase inhibitors in lung cancer. Sci Rep.

[110] Diaz LA, Bardelli A (2014). Liquid biopsies: genotyping circulating tumor DNA. J Clin Oncol.

[111] Francis G, Stein S (2015). Circulating cell-free tumour DNA in the management of cancer. Int J Mol Sci.

[112] Kerr KM, Bubendorf L, Edelman MJ, Marchetti A, Mok T, Novello S, O’Byrne K, Stahel R, Peters S, Felip E, Stahel R, Felip E, Peters S, Panel Members, and Panel Members (2014). Second ESMO consensus conference on lung cancer: pathology and molecular biomarkers for non-small-cell lung cancer. Ann Oncol.

[113] Khoo C, Rogers TM, Fellowes A, Bell A, Fox S (2015). Molecular methods for somatic mutation testing in lung adenocarcinoma: EGFR and beyond. Transl Lung Cancer Res.

[114] Gatalica Z, Feldman R, Russell K, Voss A, Reddy S (2016). 1PD Differences in expression of predictive biomarkers between primary and metastatic non-small cell lung cancer tumors. J Thorac Oncol.

[115] Sherwood J, Dearden S, Ratcliffe M, Walker J (2015). Mutation status concordance between primary lesions and metastatic sites of advanced non-small-cell lung cancer and the impact of mutation testing methodologies: a literature review. J Exp Clin Cancer Res.

[116] Srinivasan M, Sedmak D, Jewell S (2002). Effect of fixatives and tissue processing on the content and integrity of nucleic acids. Am J Pathol.

[117] Bordi P, Re M Del, Danesi R, Tiseo M (2015). Circulating DNA in diagnosis and monitoring EGFR gene mutations in advanced non-small cell lung cancer. Transl Lung Cancer Res.

[118] Dahl E, Jung A, Fassunke J, Hummel M, Penzel R, Dietmaier W, LaΔmann S (2015). [Chances and risks of blood-based molecular pathological analysis of circulating tumor cells (CTC) and cell-free DNA (cfDNA) in personalized cancer therapy: positional paper from the study group on liquid biopsy of the working group for molecular pathology in the German Society of Pathology (DGP)]. [Article in German]. Pathologe.

[119] Douillard JY, Ostoros G, Cobo M, Ciuleanu T, Cole R, McWalter G, Walker J, Dearden S, Webster A, Milenkova T, McCormack R (2014). Gefitinib treatment in EGFR mutated caucasian NSCLC: circulating-free tumor DNA as a surrogate for determination of EGFR status. J Thorac Oncol.

[120] Mok T, Wu YL, Lee JS, Yu CJ, Sriuranpong V, Sandoval-Tan J, Ladrera G, Thongprasert S, Srimuninnimit V, Liao M, Zhu Y, Zhou C, Fuerte F (2015). Detection and dynamic changes of EGFR mutations from circulating tumor DNA as a predictor of survival outcomes in NSCLC patients treated with first-line intercalated erlotinib and chemotherapy. Clin Cancer Res.

[121] Han B, Tjulandin S, Hagiwara K, Normanno N, Wulandari L, Laktionov K, Hudoyo A, Y He, Zhang YP, Wang MZ, Liu CY, Ratcliffe M, McCormack R, Reck M (2017). EGFR mutation prevalence in Asia-Pacific and Russian patients with advanced NSCLC of adenocarcinoma and non-adenocarcinoma histology: The IGNITE study. Lung Cancer.

[122] Jiang T, Ren S, Zhou C (2015). Role of circulating-tumor DNA analysis in non-small cell lung cancer. Lung Cancer.

[123] Oxnard GR, Thress KS, Alden RS, Lawrance R, Paweletz CP, Cantarini M, Yang JC, Barrett JC, Jänne PA (2016). Association between plasma genotyping and outcomes of treatment with osimertinib (AZD9291) in advanced non-small-cell lung cancer. J Clin Oncol.

[124] Sacher AG, Paweletz C, Dahlberg SE, Alden RS, O’Connell A, Feeney N, Mach SL, Janne PA, Oxnard GR (2016). Prospective validation of rapid plasma genotyping for the detection of EGFR and KRAS mutations in advanced lung cancer. JAMA Oncol.

[125] Sorensen BS, Wu L, Wei W, Tsai J, Weber B, Nexo E, Meldgaard P (2014). Monitoring of epidermal growth factor receptor tyrosine kinase inhibitor-sensitizing and resistance mutations in the plasma DNA of patients with advanced non-small cell lung cancer during treatment with erlotinib. Cancer.

[126] Normanno N, Denis MG, Thress KS, Ratcliffe M, Reck M (2017). Guide to detecting epidermal growth factor receptor (EGFR) mutations in ctDNA of patients with advanced non-small-cell lung cancer. Oncotarget.

[127] Lee G, Lee HY, Park H, Schiebler ML, van Beek EJR, Ohno Y, Seo JB, Leung A (2017). Radiomics and its emerging role in lung cancer research, imaging biomarkers and clinical management: State of the art. Eur J Radiol.

[128] Eisenhauer EA, Therasse P, Bogaerts J, Schwartz LH, Sargent D, Ford R, Dancey J, Arbuck S, Gwyther S, Mooney M, Rubinstein L, Shankar L, Dodd L (2009). New response evaluation criteria in solid tumours: revised RECIST guideline (version 1.1). Eur J Cancer.

[129] Angulo B, Lopez-Rios F, Gonzalez D (2014). A new generation of companion diagnostics: cobas BRAF, KRAS and EGFR mutation detection tests. Expert Rev Mol Diagn.

[130] Fenizia F, De Luca A, Pasquale R, Sacco A, Forgione L, Lambiase M, Iannaccone A, Chicchinelli N, Franco R, Rossi A, Morabito A, Rocco G, Piccirillo MC, Normanno N (2015). EGFR mutations in lung cancer: from tissue testing to liquid biopsy. Future Oncol.

[131] Gately K, O’Flaherty J, Cappuzzo F, Pirker R, Kerr K, O’Byrne K (2012). The role of the molecular footprint of EGFR in tailoring treatment decisions in NSCLC. J Clin Pathol.

[132] Powrózek T, Krawczyk P, Jarosz B, Mlak R, Wojas-Krawczyk K, Sawicki M, Stencel D, Trojanowski T, Milanowski J (2014). The application of real-time PCR technique to detect rare cell clones with primary T790M substitution of EGFR gene in metastases of non-small cell lung cancer to central nervous system in chemotherapy naive patients. Pathol Oncol Res.

[133] Zhao J, Feng HH, Zhao JY, Liu LC, Xie FF, Xu Y, Chen MJ, Zhong W, Li LY, Wang HP, Zhang LI, Xiao YI, Chen WJ, Wang MZ (2016). A sensitive and practical method to detect the T790M mutation in the epidermal growth factor receptor. Oncol Lett.

[134] Su KY, Chen HY, Li KC, Kuo ML, Yang JC, Chan WK, Ho BC, Chang GC, Shih JY, Yu SL, Yang PC (2012). Pretreatment epidermal growth factor receptor (EGFR) T790M mutation predicts shorter EGFR tyrosine kinase inhibitor response duration in patients with non-small-cell lung cancer. J Clin Oncol.

[135] Kim DW, Lee DH, Kang JH, Park K, Han JY, Lee JS, Jang IJ, Kim HY, Son J, Kim JH (2014). Clinical activity and safety of HM61713, an EGFR-mutant selective inhibitor, in advanced non-small cell lung cancer (NSCLC) patients (pts) with EGFR mutations who had received EGFR tyrosine kinase inhibitors (TKIs). J Clin Oncol.

[136] Wang Z, Yang JJ, Huang J, Ye JY, Zhang XC, Tu HY, Han-Zhang H, YL Wu (2017). Lung Adenocarcinoma Harboring EGFR T790M and In Trans C797S Responds to Combination Therapy of First- and Third-Generation EGFR TKIs and Shifts Allelic Configuration at Resistance. J Thorac Oncol.

[137] Arulananda S, Do H, Musafer A, Mitchell P, Dobrovic A, John T (2017). Combination Osimertinib and Gefitinib in C797S and T790M EGFR-Mutated Non-Small Cell Lung Cancer. J Thorac Oncol.

[138] Niederst MJ, Hu H, Mulvey HE, Lockerman EL, Garcia AR, Piotrowska Z, Sequist LV, Engelman JA (2015). The Allelic Context of the C797S Mutation Acquired upon Treatment with Third-Generation EGFR Inhibitors Impacts Sensitivity to Subsequent Treatment Strategies. Clin Cancer Res.

[139] Tan CS, Gilligan D, Pacey S (2015). Treatment approaches for EGFR-inhibitor-resistant patients with non-small-cell lung cancer. Lancet Oncol.

